# Spinal Anaesthesia with Hyperbaric Prilocaine in Day-Case Perianal Surgery: Randomised Controlled Trial

**DOI:** 10.1155/2014/608372

**Published:** 2014-10-14

**Authors:** Ozden Gorgoz Kaban, Dilek Yazicioglu, Taylan Akkaya, M. Murat Sayin, Duray Seker, Haluk Gumus

**Affiliations:** Ministry of Health, Diskapi Yildirim Beyazit Training and Research Hospital, Irfan Bastug Caddesi 112 Altındağ, Akasya Sokak 9/10 Koru Mah. Cayyolu, 06810 Ankara, Turkey

## Abstract

*Background*. The local anaesthetics used in day-case spinal anaesthesia should provide short recovery times. We aimed to compare hyperbaric prilocaine and bupivacaine in terms of sensory block resolution and time to home readiness in day-case spinal anaesthesia. *Methods*. Fifty patients undergoing perianal surgery were randomized into two groups. The bupivacaine-fentanyl group (Group B) received 7.5 mg, 0.5% hyperbaric bupivacaine + 20 *μ*g fentanyl in total 1.9 mL. The prilocaine-fentanyl group (Group P) received 30 mg, 0.5% hyperbaric prilocaine + 20 *μ*g fentanyl in the same volume. *Results*. Time to L1 block and maximum block was shorter in Group P than in Group B (Group P 4.6 ± 1.3 min versus Group B 5.9 ± 01.9 min, *P* = 0.017, and Group P 13.2 ± 7.5 min versus Group B 15.3 ± 6.6 min, *P* = 0.04). The time to L1 regression and S3 regression of the sensorial block was significantly shorter in Group P than in Group B (45.7 ± 21.9 min versus 59.7 ± 20.9 min, *P* = 0.024, and 133.8 ± 41.4 min versus 200.4 ± 64.8 min, *P* < 0.001). The mean time to home readiness was shorter for Group P than for Group B (155 ± 100.2 min versus 207.2 ± 62.7 min (*P* < 0.001)). *Conclusion*. Day-case spinal anaesthesia with hyperbaric prilocaine + fentanyl is superior to hyperbaric bupivacaine in terms of earlier sensory block resolution and home readiness and the surgical conditions are comparable for perianal surgery.

## 1. Introduction

The incidence of perianal surgery varies among institutions, accounting for up to 10% of general surgical procedures [1]. The procedure is suitable to perform on a day-case basis with spinal anaesthesia [2, 3]. However, prolonged sensory and motor block and urinary retention can cause a delay in discharge [4, 5].

Day-case spinal anaesthesia with short-acting local anaesthetics such as lidocaine and chloroprocaine can provide short times to discharge [6, 7]. However the association of lidocaine with transient neurologic symptoms (TNS) and chloroprocaine with neurologic injury has limited the use of these agents in spinal anaesthesia [8, 9]. Bupivacaine is safe with a very low incidence of associated TNS, but the prolonged sensory and motor block are a disadvantage for day-case spinal anaesthesia [10]. The use of small doses of bupivacaine with the addition of opioids is proposed to enhance the recovery of the spinal block [11].

The recently introduced local anaesthetic agent, hyperbaric prilocaine, has a short duration of action and the TNS incidence is low [12, 13]. Hyperbaric prilocaine provides faster spinal block onset and earlier patient recovery in ambulatory surgery compared to plain prilocaine [14]. Plain prilocaine was also compared to bupivacaine in day-case surgery and the authors concluded that bupivacaine provided shorter block duration [15]. The baricity of the local anaesthetic agent is the major factor that influences the distribution of the local anaesthetic in the subarachnoid space [16]. Scientific evidence regarding the differences of spinal block characteristics of hyperbaric prilocaine and hyperbaric bupivacaine is lacking.

This study compared 2% hyperbaric prilocaine 30 mg + fentanyl 20 *μ*g to 0.5% hyperbaric bupivacaine 7.5 mg + fentanyl 20 *μ*g for day-case spinal anaesthesia. The outcome measures were anaesthetic recovery as evaluated with the time to sensory block resolution to S3 dermatome and time to home readiness and the efficacy of the spinal block in perianal surgery.

## 2. Methods

The ethical approval for this study was provided by the Erciyes University, Faculty of Medicine Ethics Committee, Kayseri, Turkey (03.01.2012, number 2012/74, Chairperson Professor K. Kose) (Clinicaltrials.gov identifier: NCT01880775). Fifty consenting patients scheduled for perianal surgery were enrolled in this prospective randomized trial. Patients with contraindications for outpatient surgery or spinal anaesthesia, known sensitivity to the study drugs, or previous voiding difficulty, patients taking anticholinergic medications, and emergency cases were excluded from the study. The following patient parameters were recorded: gender, age, body mass index (BMI), concomitant diseases, and the American Society of Anesthesiologists (ASA) physiologic state. The patients were asked to void before surgery.

A peripheral intravenous (IV) catheter was inserted and a 7 mL kg^−1^ crystalloid infusion was initiated. The patients were premedicated with 0.03 mg kg^−1^ midazolam IV. Heart rate and peripheral oxygen saturation (SpO_2_) were monitored continuously; systolic, diastolic, and mean arterial pressure (MAP) were measured noninvasively at 5 min intervals during the procedure and at 15 min intervals during the postanaesthesia care unit (PACU) stay. The baseline values were recorded. Nasal oxygen 2 L min^−1^ was administered during the whole procedure.

The patients were randomized into two groups using a computer-generated sequence of numbers, and sealed envelopes were used for allocation. The bupivacaine-fentanyl group (Group B) (*n* = 25) received 1.5 mL (7.5 mg) 0.5% hyperbaric bupivacaine (Marcaine, heavy bupivacaine 5 mg mL 0.5%, glucose 8%, Astra Zeneca, Sweden) and 0.4 mL fentanyl (Fentanyl citrate, Abbott Pharmaceuticals, IL, USA) (20 *μ*g) in a total 1.9 mL. The prilocaine-fentanyl group (Group P) (*n* = 25) received 1.5 mL (30 mg) 0.5% hyperbaric prilocaine (Prilotekal, prilocaine 20 mg mL 2%, glucose 6%, Mercury Pharma, UK) and 0.4 mL fentanyl (20 *μ*g) in the same volume.

Spinal anaesthesia was performed at the L4-5 intervertebral space with the patient in the sitting position with a midline approach and a 25 G needle. After verifying free flow of clear cerebrospinal fluid, the prepared solution was injected into the intrathecal space in 15 seconds. The patients remained in this position for 2 minutes after the injection and were placed in the lithotomy position thereafter.

### 2.1. Intraoperative Assessment and Treatments

The sensorial block was measured at the midclavicular line with a pinprick test (via a 22 gauge hypodermic needle) at 1 min intervals until the maximum block was achieved and at 15 min intervals thereafter until the block resolved to S3 dermatome. The motor block was measured when the maximum dermatomal spread was achieved using the modified Bromage scale (0: no motor block, 1: hip blocked, 2: hip and knee blocked, and 3: hip, knee, and ankle blocked). The sensorial and motor block were evaluated by an anaesthesiologist blinded to group allocation. Motor block assessment was not done during surgery. Fentanyl (50 *μ*g), midazolam (1–5 mg), and propofol (10–20 mg incremental boluses) were used for rescue analgesia and sedation.

The time of subarachnoid injection, the onset of sensorial block (block at L1 dermatome), and the readiness for surgery (block at T10 dermatome), as well as the maximum block level and time to reach the maximum block level were recorded. Hypotension (defined as a ≥30% decrease in the systolic blood pressure in comparison with the baseline values or a systolic blood pressure of less than 80 mmHg) was treated with 250 mL crystalloid fluid boluses or 5 mg ephedrine IV. The total amount of fluid was registered. Bradycardia (defined as a heart rate ≤50 beats/min) was treated with 0.5 mg atropine IV. The periods of desaturation (SpO_2_ < 95) were recorded. The duration of surgery was defined as the time between surgical incision and wound closure.

### 2.2. Postoperative Assessment and Treatments

At the end of surgery, the patients were transferred to the PACU. Resolution of the spinal block was assessed by the time to two-segment L1 and S3 regression of the sensory block. The regression of motor block was also determined. Pain was measured with a visual analogue scale (VAS) (0: no pain; 10: maximum pain) at rest and during mobilization. Postoperative analgesia was provided with 50 mg tramadol and it was first administered when the pain score was greater than 3.

The first analgesic intake and total analgesic consumption were determined. The patients left the PACU after achieving an Aldrete score of at least 9, and the time spent in the PACU was recorded [17]. The patients were assessed for their ability to sit, stand, walk, and urinate at 15-minute intervals. The postoperative urinary retention (POUR) was evaluated at hourly intervals in the PACU and ward; ultrasonic bladder scanning was used for this purpose. If the bladder volume exceeded 500 mL and the patient had not voided spontaneously, urinary catheterization was planned. The time to home readiness was assessed as the time from the end of surgery until the patients reached a postanaesthesia discharge score (PADS) ≥9 and were able to void spontaneously or received a urinary catheter and the sensory block resolved to S3 dermatome. Any adverse events were recorded before discharge, including postoperative nausea and vomiting (PONV) or voiding difficulty. All of the patients were contacted the next day by telephone and questioned regarding pain, headache, use of analgesics, and complaints of TNS, which were defined as pain, dysesthesia, or both in the buttocks and/or lower extremities. During their control visit at the hospital 3 days after the surgery, patients also completed a questionnaire regarding headaches, TNS, and their rating of the anaesthetic method (unsatisfied, satisfied, or very satisfied).

### 2.3. Statistical Analysis

The relation between the independent parameters and the onset and recovery of the spinal block was statistically determined. SPSS for Windows version 11.5 (SPSS Inc., Chicago, IL, USA) was used. The Kolmogrov Smirnov test was used to test the normality of the distribution for continuous variables. These data were expressed as the mean ± standard deviation or median (minimum-maximum), where applicable. The mean differences were compared using an unpaired Student's *t*-test, and the Mann-Whitney *U* test was used to compare median values. The hemodynamic parameters (i.e., systolic, diastolic, and mean blood pressure, pulse rate, and oxygen saturation) were evaluated by repeated measures of ANOVA. The Greenhouse-Geisser test was applied to test the significance of the interaction term (i.e., time × group). The nominal data were analysed using Fisher's exact test or the Pearson's chi-squared test, where applicable. A *P* value less than 0.05 was considered statistically significant. For all possible multiple comparisons, the Bonferroni correction was applied to control for type I errors.

A sample size of 25 per group was required to detect at least a 30-minute difference (SD = 35.2) in S3 regression time and a 45-minute difference (SD = 38.9) in S1 regression time with a power of 90% at the 5% significance level. The differences of 30 minutes and 45 minutes were taken from literature [12, 18]. The primary outcome variables were the sensory block resolution to S3 block and time to home readiness. Other outcome variables included the onset time of the block, maximum dermatomal spread of the block, degree and motor block resolution time, length of stay in the PACU, hemodynamic parameters, and adverse events.

## 3. Results

### 3.1. Patient Characteristics

Fifty patients were enrolled in the study; none of the enrolled patients was excluded. There was no difference between treatment groups regarding age, weight, height, BMI, or gender. (Table 1). All of the operations were completed with the planned spinal anaesthesia method.

### 3.2. Onset of the Spinal Block

**Figure 1:**
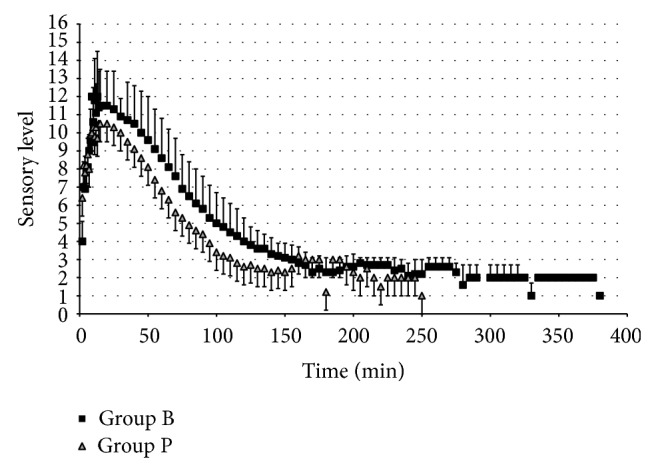
Onset and resolution of the sensory block.

The mean time to L1 block was shorter for Group P than for Group B (4.6 ± 1.3 min versus 5.9 ± 01.9 min, *P* = 0.017). The mean time to maximum sensory block was shorter for Group P than for Group B (13.2 ± 7.5 min versus 15.3 ± 6.6 min, *P* = 0.04). Maximum dermatomal spread of the block was T9 (6–12) in Group B and T9 (6–12) in Group P (*P* = 0.657). The groups were similar regarding the degree of motor block at the time that each group reached maximum sensory block (Figure 1) (Table 2).

### 3.3. Intraoperative Events and Treatments

The blood pressures and heart rates of all of the patients were stable throughout the study period. The mean MAP at the time that each group reached maximum sensory block was similar. One patient in each group experienced hypotension that needed treatment with additional fluid and ephedrine, and 1 patient in Group B experienced bradycardia and required treatment with atropine. Oxygen desaturation was not observed in any group. The groups were similar regarding intraoperative adverse events and treatments. One patient in each group required sedation. The maximum midazolam dose was 5 mg. The mean duration of surgery was similar in both groups (18 ± 28 min for Group *P* versus 20 ± 10 min for Group B).

### 3.4. Resolution of the Spinal Block

The mean time to two-segment resolution was similar between the groups (19 ± 12.4 min for Group P versus 23 ± 12.5 min for Group B (*P* = 0.214)). The mean times to L1 regression and S3 regression of the sensorial block were significantly shorter for Group P than for Group B (45.7 ± 21.9 min versus 59.7 ± 20.9 min, *P* = 0.024 (time to L1 regression), and 133.8 ± 41.4 min versus 200.4 ± 64.8 min, *P* < 0.001 (time to S3 regression)). The motor block resolved in both groups by the time the block resolved to the S3 dermatome. The length of stay in the PACU and the time required to stand and walk without assistance were different between groups, but no difference was found in the mean time to sit. The mean PACU stay was 63.2 ± 28 min for Group P and 98.8 ± 37 min for Group B (*P* < 0.001) (Figure 1) (Table 2).

### 3.5. Postoperative Events and Treatments

**Figure 2:**
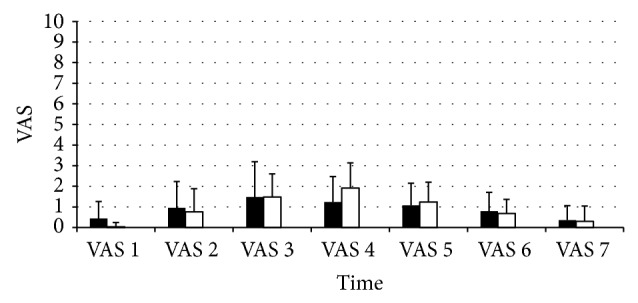
Postoperative VAS pain scores. VAS: visual analog scale.

The postoperative VAS scores for Groups P and B were similar. The mean time to first analgesic intake was 192 min for group P versus 277 min for Group B; the difference was not statistically significant (Figure 2).

The time to spontaneous voiding was also similar between the two groups. The mean time to S3 resolution of sensory block was shorter for Group P than for Group B (133.8 ± 41.4 and 200.4 ± 64.8 min, resp.) (Table 2). The groups were also similar regarding postoperative adverse events. One patient in each group had urinary retention; these patients were treated with urinary catheterization. The acceptance of the anaesthesia technique was rated as either satisfied or very satisfied by the patients; none of the patients reported to be unsatisfied. TNS were not observed in either spinal anaesthesia group at the 3-day postoperative follow-up.

## 4. Discussion

Discharge delay is a major concern in day-case spinal anaesthesia [4, 5]. This study demonstrated that spinal anaesthesia with 2% hyperbaric prilocaine 30 mg + 20 *μ*g fentanyl provides faster sensorial block resolution and earlier home readiness compared with 0.5% hyperbaric bupivacaine 7.5 mg + 20 *μ*g fentanyl. Time to block onset was also faster with the hyperbaric prilocaine-fentanyl combination and the surgical conditions were comparable to the hyperbaric bupivacaine-fentanyl combination.

Bupivacaine has been widely studied in day-case spinal anaesthesia. Lacasse et al. used 0.75% 7.5 mg bupivacaine in anorectal surgery and reported the time to S2 regression as 329 min [19]. In another study comparing different bupivacaine doses, the time to the resolution of the spinal block to the S2 dermatome with 15 mg bupivacaine was 343 min [10]. The long recovery times reported in these studies may be explained by the high concentration of bupivacaine used in the first study and the high dose bupivacaine in the second. Ben David et al. also compared different doses of bupivacaine and reported that 0.5%, 7.5 mg bupivacaine is the optimum dose for day-case anaesthesia, whereas 5 mg bupivacaine is associated with intraoperative pain [20]. Inadequate anaesthesia with low doses of bupivacaine has been reported, and the addition of an opioid seems to overcome this problem [21, 22]. Fentanyl is suitable for this purpose as it has a quick onset, medium-length lasting effect, and low risk of late-onset respiratory depression. Intrathecal 10–25 *μ*g fentanyl is safe and increases the quality of the sensory block without prolonging motor block [23, 24].

In the present study, a bupivacaine-opioid mixture, as an active control of proven efficacy, was used to evaluate the effectiveness of 30 mg hyperbaric prilocaine in day-case spinal anaesthesia for perianal surgery. Different doses of either plain or hyperbaric prilocaine have been studied in day-case spinal anaesthesia. Camponovo et al. compared the use of 40 mg and 60 mg hyperbaric prilocaine doses with 60 mg plain prilocaine in ambulatory surgery. The authors reported that hyperbaric prilocaine is superior to plain prilocaine in the ambulatory setting in terms of faster time to motor block resolution and shorter durations of surgical block [14]. The time to home discharge was reported to be 256 min with 60 mg and 208 min with 40 mg hyperbaric prilocaine; the time to home readiness was longer compared to our results but the doses used were higher and the maximum spread of the sensory block was not reported in this study. Black et al. reported that the use of 20 mg plain prilocaine and 20 *μ*g fentanyl was comparable to the use of 7.5 mg bupivacaine and 20 *μ*g fentanyl, and prilocaine provided shorter times to recovery [23]. This study defined the recovery of spinal block as the time to sensory block regression to the L4 dermatome. Further regression of the sensory block is unclear. The resolution of the sensory block to S3 must be evaluated, especially in surgery with high risk of urinary retention. As perianal surgery is associated with a high risk of urinary retention, we consider time to sensory block resolution to S3 dermatome as a better outcome.

A recent study by Gebhardt et al. compared 3 different doses of hyperbaric prilocaine in perianal surgery [18]. They reported 199, 219, and 229 minutes discharge times with 10, 20, and 30 mg hyperbaric prilocaine, respectively. The discharge time as well as the time to void with 30 mg hyperbaric prilocaine was longer compared with our results. In that study, the patients waited in the sitting position for 10 minutes after the spinal injection, this might have limited the spread of the sensory block. In our method, the patients waited in the sitting position for only 2 minutes, and the wider spread of the local anaesthetic probably resulted in a lower concentration of local anaesthetic per segment and faster elimination from the intrathecal space [25, 26]. The authors recommended 10 mg hyperbaric prilocaine for perianal outpatient surgery; however, they also noted that the procedures should be limited to the perianal skin. Despite this recommendation and since our series included perianal fistulas and surgical interventions that involved deeper tissues we preferred to use 30 mg prilocaine.

In addition to the advantage of faster block regression and early home readiness, we showed that the block quality with 30 mg hyperbaric prilocaine + 20 *μ*g fentanyl was similar to 7.5 mg hyperbaric bupivacaine + 20 *μ*g fentanyl. The maximum block height was similar with both prilocaine and bupivacaine in our study. The block height is determined by the baricity of the local anaesthetic solutions as well as the position of the patient. The hyperbaric solutions of bupivacaine and prilocaine are identical in their glucose concentration. The motor block intensity of intrathecal 7.5 mg bupivacaine is well defined. Bromage grade 2 block was reported in 53% of the patients with 7.5 mg bupivacaine and the time to complete resolution of motor block was 119 minutes [19, 20]. However data concerning 30 mg prilocaine is lacking. In the present study Bromage grade 2 motor block was observed in 48% of the patients in Group B and in 12% of the patients in Group P. Patients in Group P were significantly earlier able to walk unassisted.

Along with prolonged sensory and motor block, pain is an important cause for discharge delay [5]. Despite the shorter block duration in Group P, the postoperative VAS pain scores and the time to first analgesic intake were comparable between the groups in our study.

A recovery difference of 35 minutes is significant in the day-case setting. Decreasing the time to discharge from the PACU by more than 10% can decrease PACU congestion by 20%. Shorter PACU stays should enable PACUs to increase the number of patients served and also increase the quality of care and reduce the risk of postanaesthesia complications [27]. Hyperbaric prilocaine is more economical than bupivacaine if the PACU stay is shorter than at least 130 minutes [28].

TNS were not observed in our study population; however the study was powered to compare the recovery times of the spinal block between groups so we are not able to comment on adverse events and this is a limitation of this study.

In conclusion, day-case spinal anaesthesia with prilocaine 30 mg + 20 *μ*g fentanyl provides faster sensory block resolution and home readiness compared to 7.5 mg bupivacaine + 20 *μ*g fentanyl and the surgical conditions are comparable for perianal surgery.

## Figures and Tables

**Table 1:** Patient characteristics and surgical data.

	Group P (*n* = 25)	Group B (*n* = 25)	*P* value
Age (years)	37.8 ± 12.4	38.4 ± 13.3	0.878
Gender (*n*)			
Male	12 (48.0%)	16 (64.0%)	0.254
Female	13 (52.0%)	9 (36.0%)	
BMI (kg/m^2^)	26.4 ± 5.2	27.3 ± 5.2	0.541
ASA (*n*)			
I	15 (60.0%)	13 (52.0%)	0.569
II	10 (40.0%)	12 (48.0%)	
Operation (*n*)			0.483
Haemorrhoid	14 (56.0%)	13 (52.0%)	
Perianal fissure	10 (40.0%)	11 (44.0%)	
Perianal fistula	3 (12.0%)	1 (4.0%)	
Duration of surgery (min)	18 ± 9.4	20 ± 10	0.387

BMI: body mass index, ASA: American Society of Anaesthesiologists physiologic state. Results are presented as mean ± standard deviation, numbers, and percentages.

**Table 2:** Recovery and motor block characteristics.

	Group P	Group B	*P*
PACU time (min)	63 ± 28	99 ± 37	<0.001∗
Time to sit unassisted (min)	30.6 ± 11	36.1 ± 13.7	0.109
Time to stand unassisted (min)	138.7 ± 55.9	172.2 ± 85.2	0.002∗
Time to walk unassisted (min)	136.9 ± 53.6	172.0 ± 82.5	0.002∗
Time to void (min)	152.8 ± 104.8	172.4 ± 130.8	0.682
Recovery time (time to S3 resolution of sensory block) (min)	133.8 ± 41.4	200.4 ± 64.8	<0.001∗
Time to home readiness (min)	155 ± 100.2	207.2 ± 62.7	<0.001∗
Bromage score at max. block 0/1/2/3 (*n*)	13/6/3/3	8/1/12/4	0.152
Bromage score at 1 hour 0/1/2/3 (*n*)	22/2/1/0	11/9/4/1	<0.001∗
Bromage score at 2 hours 0/1/2/3 (*n*)	25/0/0/0	22/3/0/0	0.235

Bromage score: 0: no motor block, 1: hip blocked, 2: hip and knee blocked, 3: hip, knee, and ankle blocked, and *n*: number of patients with each degree of motor block at the corresponding time. Data are presented as mean ± standard deviation and numbers.

∗Significant difference between groups.
