# Correction to “Spinal Anaesthesia With Hyperbaric Prilocaine in Day‐Case Perianal Surgery: Randomised Controlled Trial”

**DOI:** 10.1155/tswj/9829150

**Published:** 2025-12-28

O. Gorgoz Kaban, D. Yazicioglu, T. Akkaya, M. Murat Sayin, D. Seker, and H. Gumus, “Spinal Anaesthesia With Hyperbaric Prilocaine in Day‐Case Perianal Surgery: Randomised Controlled Trial,” *The Scientific World Journal*, 2014, https://doi.org/10.1155/2014/608372

In the article, a reference to a retracted article [1] was included in error. The authors have requested to remove the reference and correct the following sentence:

“Day‐case spinal anaesthesia with short‐acting local anaesthetics such as lidocaine can provide short times to discharge [6, 7].”

should read

“Day‐case spinal anaesthesia with short‐acting local anaesthetics such as lidocaine can provide short times to discharge [6].”

We apologize for this error.
